# Hepatitis C virus core protein-induced myeloid-derived suppressor cells promote hepatic fibrosis by regulating hepatic stellate cell function via TGF-β

**DOI:** 10.3389/fimmu.2026.1795273

**Published:** 2026-03-18

**Authors:** Fangzhuo Zhu, Shixing Zhao, Yunqi Zhang, Chengwei Tan, Jing Zhang, Qianqian Zhang

**Affiliations:** 1 School of Clinical Medicine (Affiliated Hospital), Jining Medical University, Jining, China; 2 Department of Critical Care Medicine, Affiliated Hospital of Jining Medical University, Jining, China; 3 School of Forensic Medicine, Jining Medical University, Jining, Shandong, China

**Keywords:** HCV core protein, hepatic stellate cells, liver fibrosis, myeloid-derived suppressor cells, TGF-β

## Abstract

**Background and objective:**

Myeloid-derived suppressor cells (MDSCs) constitute a population of cells with immunosuppressive functions, potentially playing a pivotal role in the progression of chronic hepatitis C (CHC) to liver fibrosis. This study aimed to elucidate the molecular mechanisms by which HCVc-induced MDSCs interact with hepatic stellate cells to influence the onset and progression of liver fibrosis.

**Methods:**

CD14^+^ monocytes were isolated and purified from healthy human peripheral blood. These cells were stimulated *in vitro* with hepatitis C virus core protein (HCVc) to induce differentiation into MDSCs. CD14^+^ monocytes or HCVc-induced MDSCs were co-cultured with the human hepatic stellate cell line LX2 to establish an *in vitro* co-culture system. The TGF-β signaling pathway was blocked using a neutralizing antibody. LX2 proliferation was assessed via the MTT assay, LX2 activation via ELISA, and LX2 apoptosis via flow cytometry.

**Results:**

HCVc induced CD14^+^ monocytes to differentiate into MDSCs. HCVc-induced MDSCs promoted LX2 proliferation and type I collagen (Col-1) synthesis while inhibiting LX2 apoptosis, thereby driving the onset and progression of liver fibrosis. This effect was suppressed by TGF-β neutralizing antibodies.

**Conclusion:**

HCVc-induced MDSCs mediate the regulation of LX2 proliferation, activation, and apoptosis via TGF-β signaling, thereby promoting hepatic fibrosis. This “HCVc–MDSCs–TGF-β–LX2” axis establishes, for the first time, a direct link between viral infection, immunosuppressive myeloid cells, and the hepatic fibrosis process, providing potential targets for developing novel therapeutic strategies for hepatitis C-associated liver fibrosis.

## 1 Introduction

Hepatitis C virus infection remains a leading cause of chronic liver disease worldwide. Currently, approximately 50 million people globally are living with chronic hepatitis C virus (HCV) infection, with around one million new cases of chronic hepatitis C occurring annually (1). The timely administration of direct-acting antivirals (DAAs) offers the opportunity for complete eradication of the HCV (2). However, HCV can successfully evade immune clearance within the host, leading to the development of chronic hepatitis in the majority of infected individuals. Without prompt treatment, this progression may result in liver fibrosis, a critical stage in the advancement to cirrhosis or hepatocellular carcinoma (3, 4). Its primary pathological feature is the excessive deposition of extracellular matrix (ECM) (5), with hepatic stellate cells (HSCs) serving as the principal source cells for ECM within fibrotic liver tissue (6).When the disease progresses to the decompensated stage of cirrhosis, patients may develop numerous critical complications that can prove life-threatening. Epidemiological studies indicate that approximately two million people worldwide die annually from severe liver disease, with one million fatalities attributable to complications of cirrhosis and another million resulting from viral hepatitis and hepatocellular carcinoma (7). Currently, several anti-fibrotic agents have entered preclinical research and clinical trial phases. However, their efficacy remains limited and they exhibit significant side effects (8). Consequently, investigating the fundamental causes of hepatic fibrosis and exploring methods to inhibit the progression of chronic hepatitis to fibrosis and cirrhosis holds considerable clinical application value.

Liver fibrosis arises from the combined effects of persistent liver injury and dysregulation of tissue repair mechanisms. During acute inflammation and liver injury, tissue repair mechanisms and anti-fibrotic responses typically maintain a dynamic equilibrium, inducing myofibroblast inactivation or apoptosis to prevent excessive fibrotic accumulation. However, in chronic liver disease progression, HSCs undergo sustained activation and transformation into myofibroblasts, leading to substantial synthesis and secretion of ECM, ultimately driving the onset and progression of liver fibrosis (9). Research indicates that during liver injury, TGF-β secreted by hepatocytes or sinusoidal endothelial cells binds to its receptor, inducing phosphorylation of Smad2/3. Subsequently, phosphorylated Smad2/3 assembles with Smad4 into a complex, translocates to the nucleus, and directly initiates transcription of fibrosis-related target genes such as collagen genes. This drives abnormal deposition of the extracellular matrix (ECM), thereby promoting liver fibrosis (10).

In recent years, the role of the hepatic immune microenvironment in the fibrotic process has garnered increasing attention. Myeloid-derived suppressor cells (MDSCs) constitute a heterogeneous population of myeloid cells generated under pathological conditions such as inflammation, tumorigenesis, and autoimmune diseases, arising from impaired differentiation of immature myeloid cells. Upon activation, they accumulate in peripheral and target tissues, suppressing immune responses through multiple mechanisms—particularly by impairing T-cell function—thereby fostering an immunosuppressive state (11). MDSCs are primarily categorized into two major subpopulations: monocytic myeloid-derived suppressor cells (M-MDSCs) and granulocytic myeloid-derived suppressor cells (G-MDSCs). In addition, a small population of myeloid progenitor cells with MDSC-like characteristics, termed “early MDSCs,” has also been identified in humans (12). Different subtypes of MDSCs participate in immune suppression through distinct mechanisms. M-MDSCs primarily produce substantial amounts of nitric oxide, arginase-1, and immunosuppressive cytokines including IL-10 and TGF-β to inhibit T cell activation, whereas G-MDSCs predominantly generate abundant reactive oxygen species (ROS) and inactivate T cells via close intercellular contacts (13). Recent studies have revealed that MDSCs abnormally proliferate within the chronic liver disease microenvironment and contribute to disease progression through their immunosuppressive functions. In patients with chronic HCV infection, the mean peripheral blood MDSC count exceeds that of healthy controls. HCVc can induce MDSC production in peripheral blood mononuclear cells (PBMCs) via the TLR2/PI3K/AKT/STAT3 signaling pathway and through autocrine signaling regulation (14, 15). However, systematic investigations into how HCVc-regulated MDSCs mediate HCV-associated liver fibrosis remain lacking. Therefore, comprehensive research is required to elucidate the mechanisms by which MDSCs influence liver fibrosis, thereby guiding clinical strategies for preventing and managing its progression.

The hepatitis C virus core protein (HCVc) is a structural protein that encapsulates the HCV genomic RNA and assembles into a nucleocapsid. It exerts regulatory effects on host cell apoptosis, pro-inflammatory/anti-inflammatory modulation, alterations in TLR signaling pathways, and the activation and differentiation of immune cells (16).In this study, we employed HCVc to simulate hepatitis C virus infection and discovered that HCVc-induced M-MDSCs promote the proliferation and activation of hepatic stellate cells while inhibiting their apoptosis. We further explored the potential mechanisms underlying these effects.

## 2 Materials and methods

### 2.1 Reagents

Recombinant HCV core antigen (amino acids 2-192) was purchased from Meridian Life Science, Inc. (Memphis, Tennessee, USA). Human peripheral blood lymphocyte lysate was purchased from tbd science.com (Tianjin, China). CD14 beads were purchased from Miltenyi Biotec Inc. (California, USA). PE-conjugated anti-human CD14, PE/Cy7-conjugated anti-human CD14, and PerCP-conjugated anti-human HLA-DR were purchased from BD Biosciences (New Jersey, USA). TGF-β inhibitor was purchased from MedChemExpress (New Jersey, USA). The MTT cell proliferation and cytotoxicity assay kit was purchased from Beyotime Biotech Inc (Shanghai, China). The Annexin V-FITC/PI dual-stain apoptosis assay kit was purchased from Pricella (Wuhan, China). The human type I collagen (Col-I) ELISA research kit was purchased from MEIMIAN (Wuhan, China).

### 2.2 Peripheral blood

Peripheral blood samples were sourced from clinical resources within the Health Examination Centre, Department of Gastroenterology, Department of Infectious Diseases, and Department of Hepatobiliary and Pancreatic Surgery at Jining Medical University Affiliated Hospital. Informed consent was obtained in accordance with their protocols.

### 2.3 Isolation, purification and detection of CD14^+^ monocytes

PBMCs were isolated by density gradient centrifugation (Ficoll method), and CD14^+^ cells were separated using CD14 magnetic beads (17). Cells were stained for 30 minutes with PE-conjugated anti-human CD14 antibody and PE/Cy7 or PerCP-conjugated anti-human HLA-DR antibody, following the manufacturer’s protocol (BD Bioscience). Following staining, cells were washed in staining buffer and resuspended in PBS containing 1% paraformaldehyde. Finally, cells were analyzed using a flow cytometer (Beckman Coulter CytoFLEX). Acquired data were analyzed using FlowJo 10.8.1, with purity consistently ranging between 90–95%.

### 2.4 Differentiation of CD14^+^ monocytes into myeloid-derived suppressor cells and detection

Isolated and purified CD14^+^ monocytes were added to complete RPMI 1640 medium containing 10% foetal bovine serum and 100 IU/mL penicillin and streptomycin. The mixture was transferred to a 96-well plate, with different concentrations of HCVc (1, 5, 10, 20, and 50 μg/ml) and 0.3 × 10^6^ cells/well added to each well, at a final volume of 250 μl per well. The cells were then incubated at 37 °C in a 5% CO_2_ incubator. After incubation, the cells were harvested and analyzed by flow cytometry to assess MDSC differentiation.

### 2.5 LX-2 cell culture

Human liver xanthoma cells (LX2), catalogue number: CL-0560, procured from Pricella (Wuhan, China), were cultured in high-glucose DMEM containing 10% foetal bovine serum and 1% penicillin and streptomycin, and gradually adapted to complete RPMI 1640 medium.

### 2.6 Co-culture of CD14^+^ monocytes/MDSCs with LX2 cells

Take a 24-well culture plate and place the Transwell co-culture chamber (0.4 micron) within it. Using 5 × 10^4^ cells as the baseline, add 200 μl of CD14^+^ monocyte or MDSC cell suspension to the upper chamber and 700 μl of LX2 cell suspension to the lower chamber in a 2:1 ratio. Incubate at 37 °C in a 5% CO_2_ incubator.

### 2.7 Cell proliferation assay

Following co-culture, transfer the LX2 cells from the lower chamber to a 96-well plate. Assess LX2 proliferation using the MTT cell proliferation and cytotoxicity assay kit, following the manufacturer’s protocol. Measure the absorbance of each well at 570 nm using a microplate reader (BioTek Cytation 5).

### 2.8 Collagen I ELISA assay

Detection was performed using the Type I Collagen (Col-I) ELISA research kit. Following co-culture, the supernatant from the LX2 cells was collected and tested according to the manufacturer’s instructions. The absorbance per well was measured at 450 nm wavelength using an enzyme-linked immunosorbent assay reader (BioTek Cytation 5).

### 2.9 Flow cytometry analysis of cell apoptosis

Following co-culture, LX2 cells were harvested from the lower chamber and stained according to the protocol of the Annexin V-FITC/PI dual-stain apoptosis assay kit. The stained cells were analyzed by flow cytometry, with the collected data processed using FlowJo 10.8.1.

### 2.10 Data analysis

All statistical analyses were performed using GraphPad Prism version 10.1.2 software (GraphPad Prism Software Inc., California, USA).Differences between two groups were assessed using an unpaired two-tailed Student’s t-test. For comparisons among multiple groups, one-way analysis of variance (ANOVA) followed by Tukey’s *post-hoc* test was employed. A P-value of less than 0.05 was considered statistically significant.

## 3 Result

### 3.1 Elevated levels of MDSC cells and serum TGF-β in chronic HCV patients

**Figure 1:**
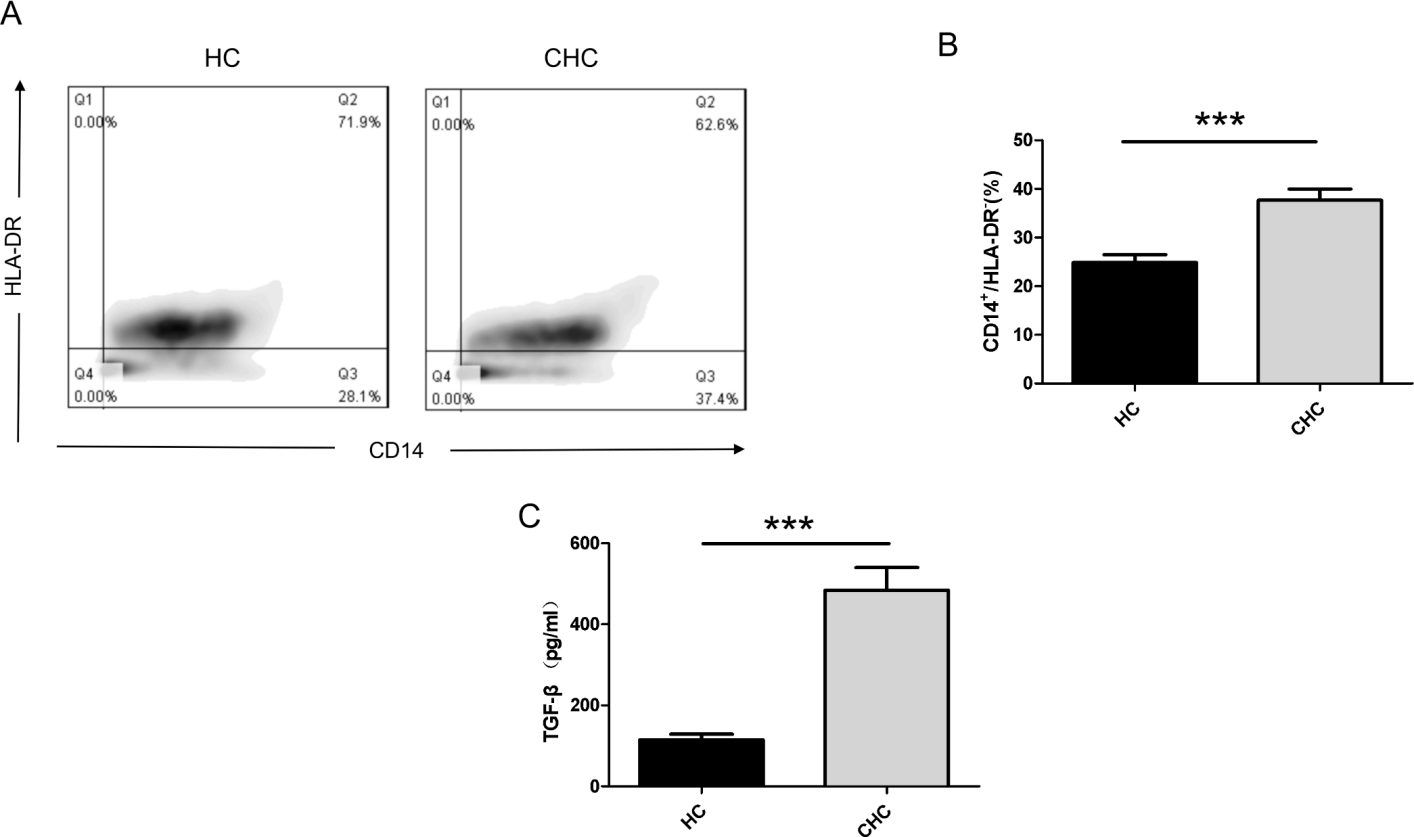
Comparison of peripheral blood CD14+HLA-DR-/low monocyte proportion and TGF-β levels between CHC and HC groups. **(A)** CD14^+^ monocytes were isolated and purified from CHC patients and healthy donors using magnetic bead sorting technology. Specific antibody staining followed by flow cytometric analysis determined the proportion of CD14+HLA-DR-/low phenotype MDSCs. **(B)** Statistical analysis of CD14^+^HLA-DR-/^low^ cells in 10 healthy donors and 10 patients with chronic hepatitis C. **(C)** ELISA detection of TGF-β expression differences in serum between the CHC and HC groups. Each experiment was performed with samples from ten independent donors (n = 10). Error bars represent standard errors of the mean. ***P < 0.001.

To validate the role of MDSCs in the chronicization of hepatitis C virus infection, we compared the proportion of MDSCs in peripheral blood between chronic hepatitis C (CHC) patients and healthy blood donors. We isolated and purified CD14^+^ monocytes via magnetic bead sorting and analyzed the proportion of MDSCs using flow cytometry. In our analyzed samples, CHC patients exhibited a distinct CD14^+^HLA-DR^-/low^ phenotype MDSC population, with a proportion significantly different from the healthy control (HC) group (**Figures 1A, B**). We employed ELISA to detect differences in TGF-β expression in peripheral blood serum from HCV patients and healthy individuals. Results revealed markedly elevated peripheral blood TGF-β levels in the CHC group compared to the HC group (**Figure 1C**).

### 3.2 HCVc induces the differentiation of CD14^+^ monocytes into MDSCs

**Figure 2:**
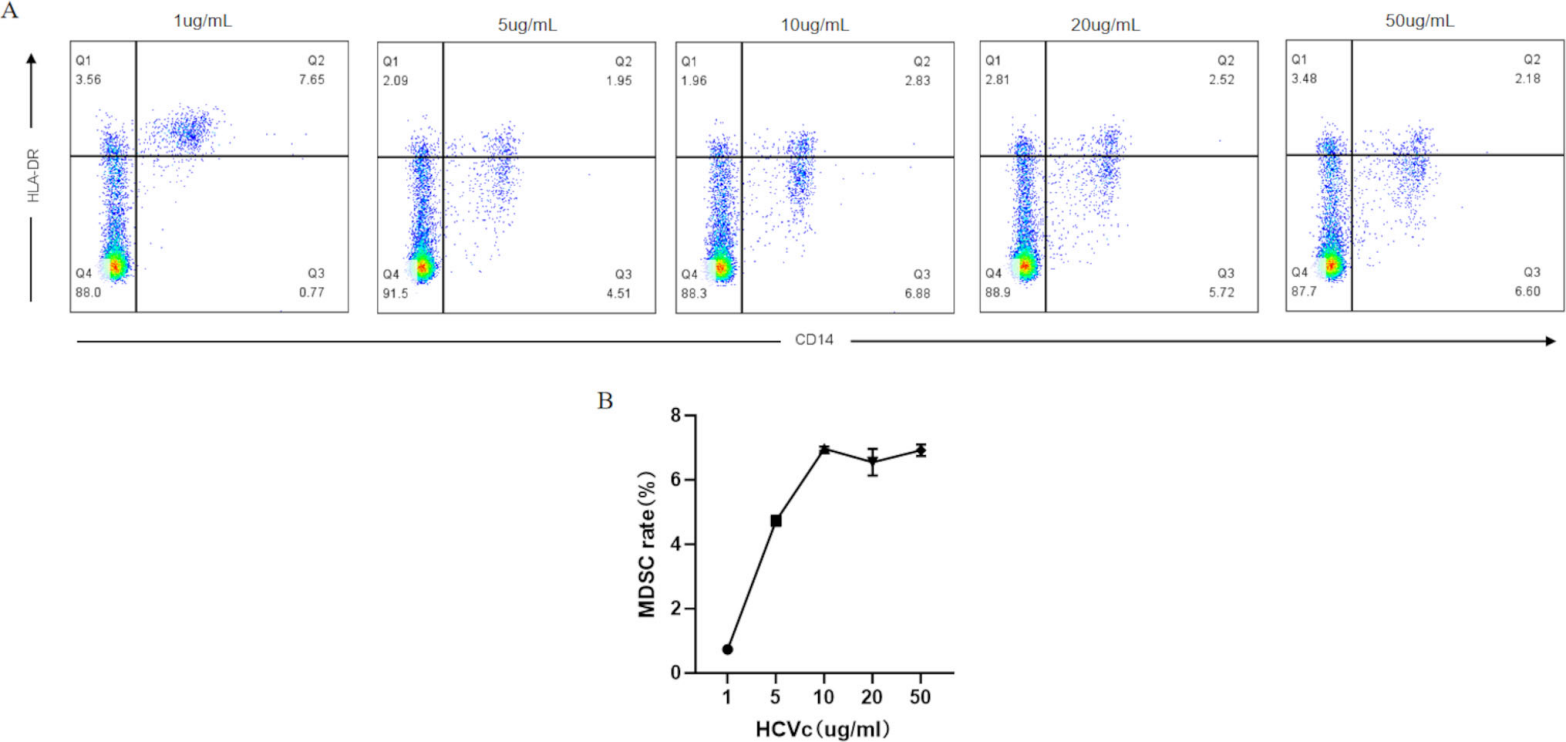
HCV core protein (HCVc) induces an MDSC-like phenotype in PBMCs. CD14^+^ monocytes were isolated and stimulated with HCVc for two days. Flow cytometry detected the expression of CD4^+^HLA-DR-/^low^ phenotype MDSCs. **(A)** Optimal stimulation concentration for inducing monocytes into MDSCs was screened via flow cytometry; **(B)** Proportion of MDSCs in CD14^+^ monocytes treated with varying doses of HCVc for 2 days, analyzed by flow cytometry. Data are representative of three independent experiments (n = 3). Error bars represent standard error of the mean.

We employed HCVc to simulate HCV infection, stimulating CD14^+^ monocytes with varying concentrations (1 μg/ml, 5 μg/ml, 10 μg/ml, 20 μg/ml, 50 μg/ml) of HCVc for 48 hours. We observed that HLA-DR expression was significantly downregulated in CD14^+^ monocytes exposed to HCVc, leading to their differentiation into CD14^+^ HLA-DR^-/low^ phenotype MDSCs, with the optimal stimulation concentration being 10 μg/ml (**Figure 2**).

### 3.3 HCVc-induced myeloid-derived suppressor cells promote LX2 proliferation and activation while inhibiting LX2 apoptosis

**Figure 3:**
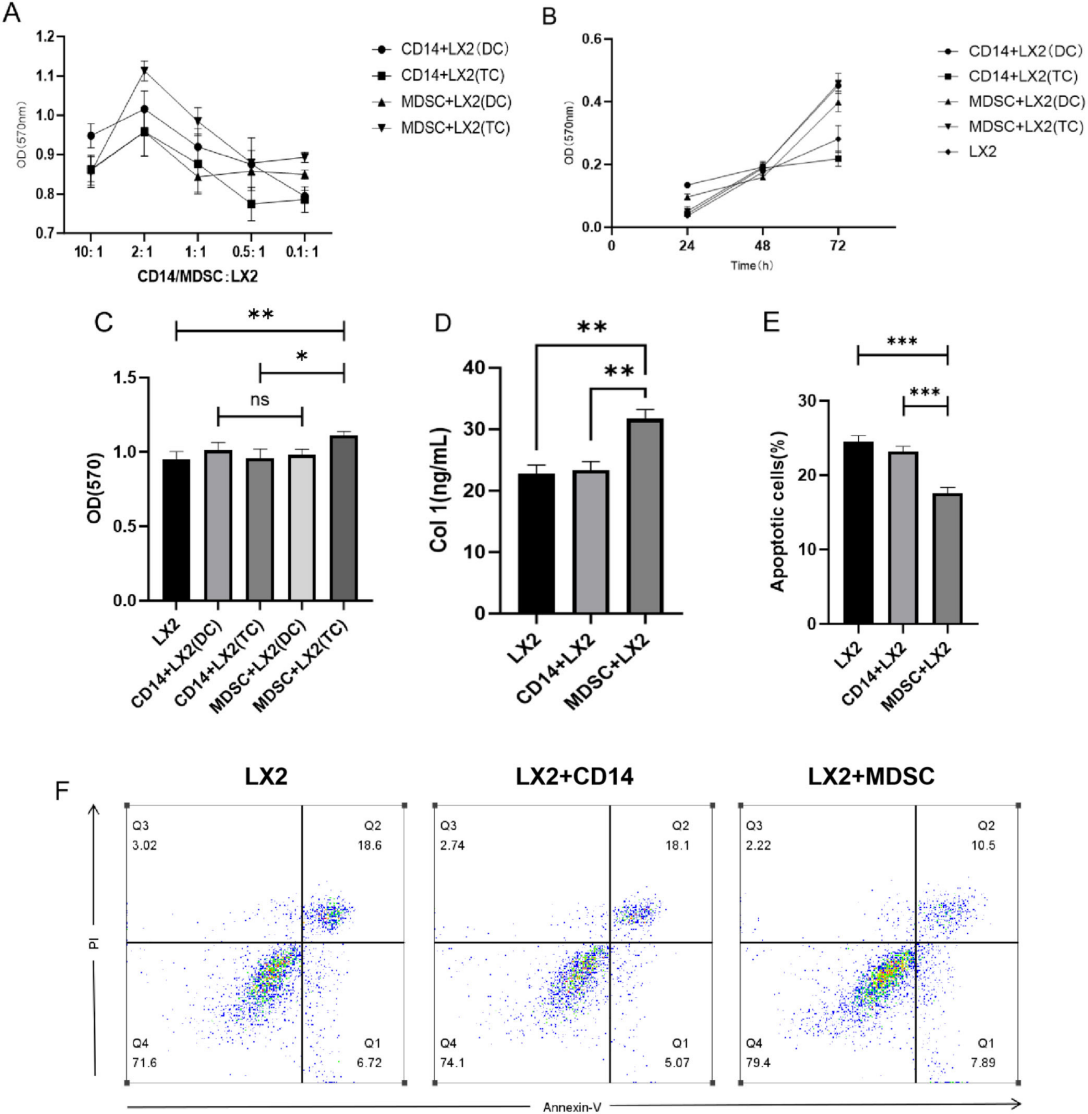
Regulation of LX2 proliferation, activation, and apoptosis by HCVc-induced MDSCs. HCVc stimulated purified CD14+ monocytes for 48 hours (CD14^+^ monocytes served as negative control group), inducing their differentiation into MDSCs. Following determination of the optimal co-culture cell ratio and duration (CD14^+^/MDSC: LX2 at 2:1, 72 hours), a co-culture system was established between CD14^+^/MDSCs and LX2 hepatocytes. **(A)** LX2 proliferation at varying CD14+/MDSC: LX2 co-culture ratios. **(B)** LX2 proliferation under different co-culture durations with CD14^+^/MDSCs. **(C)** LX2 proliferation assessed by MTT assay. **(D)** Col-1 synthesis in the co-culture system measured by ELISA. **(E)** Apoptosis in LX2 cells assessed by Annexin V/PI double staining via flow cytometry. This represents the statistical analysis of three LX2 apoptosis experiments. **(F)** A representative flow cytometric plot illustrating LX2 apoptosis. All experiments were performed in triplicate with three independent biological replicates (n = 3). Error bars denote standard errors of the mean. *P < 0.05, **P < 0.01, ***P < 0.001. DC, Direct co-culture; TC, Indirect co-culture.

To investigate the effects of MDSCs on LX2 cells, we employed direct or indirect co-culture of CD14^+^ monocytes or induced mature MDSCs with LX2 cells. The optimal co-culture concentration and duration were determined using the MTT assay. We observed that the most pronounced LX2 proliferation occurred when co-cultured at a CD14^+^/MDSCs: LX2 ratio of 2:1 for 72 hours (**Figures 3A, B**). Under these conditions, we established a co-culture system and assessed MDSC effects on LX2 proliferation via MTT assay (**Figure 3C**). Results demonstrated that indirect co-cultured MDSCs promoted LX2 proliferation (P < 0.05), whereas direct co-culture had no effect (P > 0.05). Moreover, compared to the CD14^+^+LX2 group, the MDSC+LX2 group exhibited a higher LX2 proliferation rate, with a statistically significant difference between the two groups (P < 0.05). This suggests that HCVc-induced MDSCs can promote LX2 proliferation. An ELISA assay was performed to detect type I collagen (Col-1) synthesis in the co-culture supernatant (**Figure 3D**). We observed significantly higher Col-1 synthesis in the MDSC+LX2 group than in the CD14^+^+LX2 group (P < 0.05), indicating MDSCs promote LX2 activation. Apoptosis in LX2 cells within the co-culture system was assessed via Annexin V/PI double staining and flow cytometry (**Figures 3E,F**). The results showed that LX2 apoptosis was significantly lower in the MDSC+LX2 group compared to the CD14^+^+LX2 group, with a statistically significant difference between the two groups (P < 0.05). This indicates that HCVc-induced MDSCs can promote LX2 proliferation and activation while inhibiting LX2 apoptosis.

### 3.4 HCVc-induced myeloid-derived suppressor cells regulate LX2 function via TGF-β signaling

**Figure 4:**
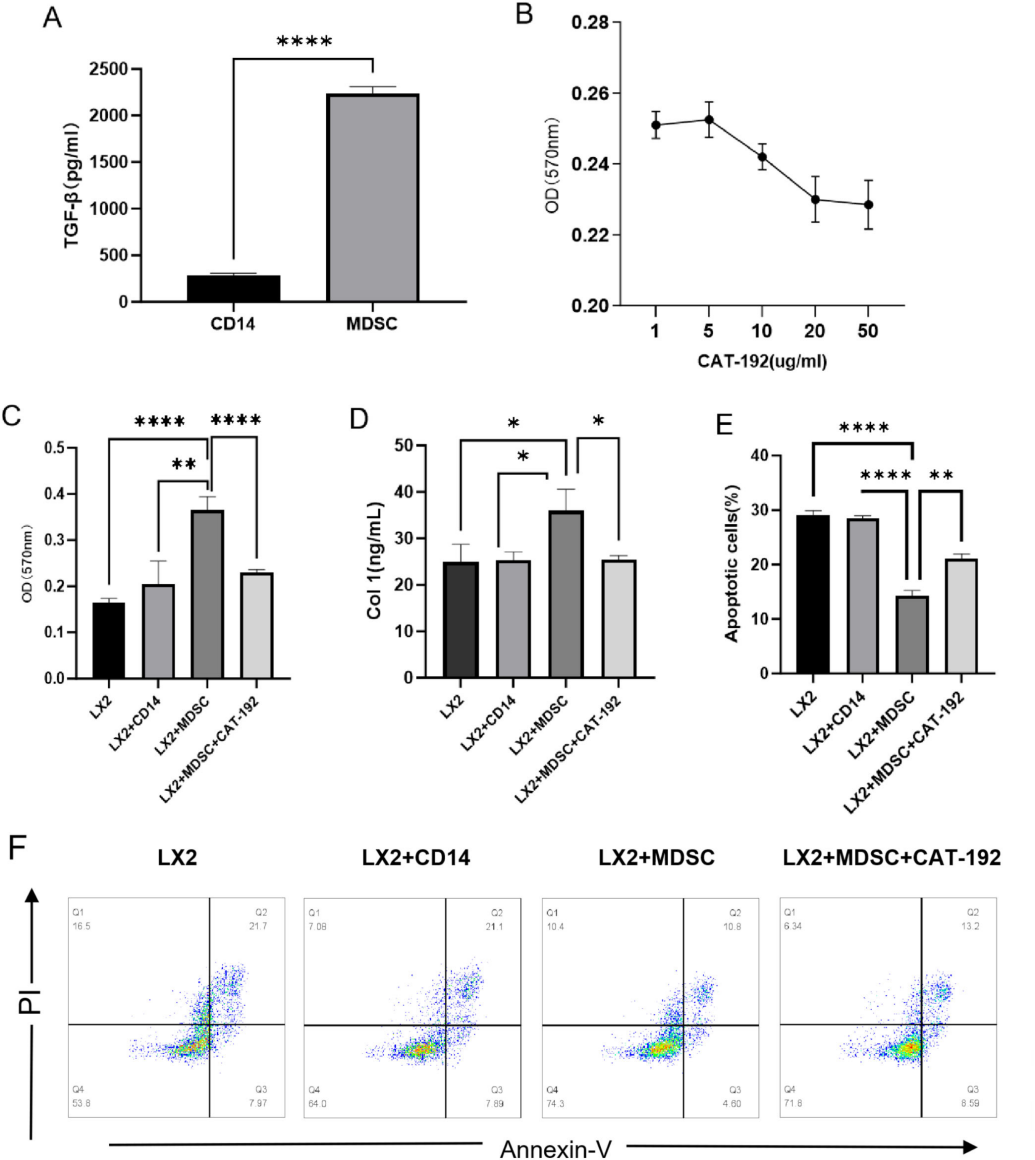
MDSCs regulate LX2 proliferation, apoptosis and activation via TGF-β signaling. As shown, TGF-β expression was markedly higher in the MDSC group than in the CD14^+^ monocyte group. MDSCs were co-cultured with LX2 cells for three days with or without TGF-β inhibitor. **(A)** ELISA detection of TGF-β differences between MDSC and CD14^+^ monocyte groups. **(B)** Regulation of LX2 by MDSCs at different concentrations of CAT-192. **(C)** MTT assay for LX2 proliferation. **(D)** ELISA detection of LX2 Col-1 secretion. **(E)** Flow cytometry analysis of LX2 apoptosis. **(F)** Representative flow cytometric analysis of LX2 apoptosis. Each condition was tested in three independent experiments (n = 3). Error bars denote standard error of the mean. *P<0.05, **P<0.01, ***P<0.001, ****P<0.0001.

Multiple cytokines can activate hepatic stellate cells (HSCs), with TGF-β1 being the most potent pro-fibrotic cytokine (18). It has been established that Kupffer cells, sinusoidal endothelial cells, and numerous other cell types possess the capacity to release TGF-β1 (19). We stimulated CD14^+^ monocytes with HCVc for 48 hours (unstimulated CD14+ cells served as negative controls) to induce their differentiation into MDSCs. The differences in TGF-β expression in the supernatants were detected using an ELISA assay (**Figure 4A**). Results demonstrated significantly higher TGF-β expression in the MDSC group compared to the CD14^+^ monocyte group, with statistical significance (P<0.05). This indicates that HCVc-induced MDSCs secrete substantial amounts of TGF-β, consistent with prior *in vivo* studies showing elevated TGF-β levels in HCV patient serum compared to healthy controls.

To further determine whether TGF-β signaling is a key regulator of LX2 function, we performed 72-hour indirect co-cultures of HCVc-induced MDSCs with LX2 cells (2:1 ratio), incorporating neutralizing antibodies against TGF-β (CAT-192) at varying concentrations into the culture system. LX2 proliferation was assessed using the MTT assay to determine the optimal inhibitory concentration of CAT-192. Results indicated that CAT-192 concentrations of 20 μg/ml and 50 μg/ml exhibited the strongest inhibitory effects on LX2 proliferation (**Figure 4B**). Based on these inhibitory effects, 20 μg/ml CAT-192 was incorporated into subsequent culture systems. LX2 proliferation was assessed via MTT assay, while apoptosis was evaluated using Annexin V/PI double staining followed by flow cytometry. LX2 activation was monitored by measuring Col-1 secretion via ELISA. We observed that inhibition of TGF-β signaling factors markedly reduced the effects of MDSCs on LX2 proliferation, activation, and apoptosis. This indicates that the promotion of LX2 proliferation and activation, as well as the suppression of LX2 apoptosis by MDSCs, occurs via TGF-β signaling (as shown in **Figures 4C–F**).

## 4 Discussion

HCV exhibits a high propensity for chronicity and is more likely than other types of hepatitis to progress to liver fibrosis and cirrhosis. Statistics indicate that as many as 75% to 85% of HCV-infected individuals will develop chronic HCV, with 10% to 20% of chronic carriers progressing to cirrhosis following 20 to 30 years of active liver disease (3). The application of direct-acting antivirals (DAAs) has markedly increased the clearance rate of HCV (20), and spared most patients from the significant side effects previously associated with interferon therapy (21). However, fibrotic activity persists, and the progression of liver fibrosis does not cease with the clearance of the hepatitis C virus (22). This study suggests that even after viral clearance, HCVc-pre-induced MDSCs and the pro-fibrotic microenvironment they establish may persist. This provides a novel mechanistic basis for explaining why fibrosis remains difficult to reverse in some patients following direct-acting antiviral (DAA) therapy. Consequently, targeting MDSCs or their downstream TGF-β signaling pathway may serve as an adjunct strategy to DAA treatment for delaying or even reversing liver fibrosis.

To further elucidate the structural basis of HCVc-mediated MDSC induction, we analyzed the molecular interactions between HCVc and MDSC precursors. Studies have demonstrated that HCVc interacts with Toll-like receptor 2 (TLR2) on the surface of monocytes, leading to the activation of the PI3K/AKT/STAT3 signaling pathway, which is critical for MDSC differentiation (23). Furthermore, structural predictions suggest that the core protein’s lipid-modified N-terminus facilitates its binding to myeloid cell membranes, enhancing its immunomodulatory effects (24). These findings provide a structural rationale for our observation that HCVc efficiently drives CD14+ monocytes toward an MDSC phenotype. This interaction likely primes the immunosuppressive microenvironment that subsequently promotes fibrogenesis.

The role of MDSCs in chronic liver disease is attracting increasing attention, as they drive inflammation, immune evasion and fibrosis (25). Our findings extend this paradigm to the field of HCV-associated liver disease. Mechanistically, MDSCs are extensively induced during HCV infection, not only contributing to viral immune evasion but also directly acting upon HSCs through sustained TGF-β secretion. This disrupts the equilibrium between activation and apoptosis, leading to excessive ECM deposition. This provides a cellular and molecular explanation for the previously observed phenomenon that “HCV infection promotes fibrosis”. The HCV core protein drives peripheral blood mononuclear cell differentiation into granulocyte-derived myeloid suppressor cells via the IL-10/STAT3 signaling pathway. while simultaneously suppressing interferon-γ production in CD4^+^ and CD8^+^ T cells through NADPH oxidase and reactive oxygen species (ROS) generation, thereby supporting sustained viral replication (26). In this study, we demonstrated that HCVc can induce CD14^+^ monocytes to differentiate into MDSCs, and that HCVc-induced MDSCs exacerbate hepatic fibrosis. Overall, the accumulation of MDSCs during HCV infection may represent a key immunosuppressive mechanism contributing to persistent HCV infection and its progression towards fibrosis. To functionally validate the role of TGF-β signaling in MDSC-mediated LX2 regulation, we employed a TGF-β neutralizing antibody (CAT-192) in our co-culture system. Our results demonstrate that blocking TGF-β signaling significantly abrogated MDSC-induced LX2 proliferation and activation, while restoring LX2 apoptosis. These findings align with previous studies showing that TGF-β is a master regulator of HSC activation and that its inhibition can attenuate fibrotic processes (27). Moreover, TGF-β signaling has been shown to synergize with inflammatory mediators to perpetuate HSC activation in chronic liver disease (28). In the context of HCV infection, TGF-β has been implicated in both immune suppression and fibrogenesis, with MDSCs serving as a critical source of this cytokine (29). Additionally, recent evidence suggests that MDSC-derived TGF-β not only activates HSCs but also creates a feedback loop that sustains MDSC expansion, further exacerbating fibrosis (30). Our study provides direct experimental evidence that HCVc-induced MDSCs rely on TGF-β signaling to modulate HSC function, thereby establishing a mechanistic link between viral proteins, immunosuppressive cells, and fibrogenesis.

Moreover, as an organ possessing immune tolerance characteristics, the liver’s microenvironment readily recruits and retains MDSCs (31). These MDSCs can be recruited to the liver under the influence of various chemokines to exert their effects. Some studies indicate that HSCs can induce peripheral blood mononuclear cells to differentiate into MDSCs (32), and can also induce MDSCs to migrate into hepatocellular carcinoma tissue (33). Through the IL-6 signaling pathway, they enhance the immunosuppressive function of MDSCs within hepatocellular carcinoma tissue, thereby promoting disease progression (34). Consequently, the interaction between HSCs and MDSCs influences the onset and development of liver disease, with MDSCs potentially representing a key factor in the susceptibility of HCV-infected individuals to liver fibrosis. To validate our hypothesis, we established a co-culture system between HCV core protein-induced MDSCs and LX2 hepatocytes. As anticipated, MDSCs promoted LX2 proliferation and activation while downregulating LX2 apoptosis. Mechanistically, the TGF-β signaling pathway constitutes a pivotal pathway in liver fibrosis, with its classical Smad-dependent pathway serving as a primary regulator of hepatic stellate cell activation (35). This study demonstrated through *in vitro* and *in vivo* experiments that HCVc-induced MDSCs significantly promote the release of TGF-β factors. Notably, to validate the relationship between MDSCs’ pro-fibrotic effects and TGF-β factors, intervention with TGF-β signaling inhibitors markedly suppressed the pro-fibrotic activity of MDSCs, further confirming the pivotal role of TGF-β signaling in this process. This indicates that HCVc-induced MDSCs provide a sustained source of TGF-β, thereby creating a favourable microenvironment for fibrotic progression. This discovery provides direct theoretical and experimental rationale for developing targeted therapies, such as inhibiting MDSC recruitment, blocking their TGF-β secretion, or disrupting downstream signaling pathways.

The present study has several methodological and conceptual limitations that should be addressed in future research to corroborate and extend our findings. Firstly, this study primarily relied on an *in vitro* co-culture system, which cannot fully recapitulate the complex hepatic microenvironment *in vivo*. Therefore, the “HCVc--MDSCs--TGF-β--LX2” axis identified in this study requires further validation using animal models of liver fibrosis. Secondly, we did not perform in-depth functional subtyping of MDSC populations. Future studies should employ single-cell sequencing and flow cytometric sorting to elucidate the specific roles of M-MDSCs and G-MDSCs in liver fibrosis progression. Thirdly, although we demonstrated the critical role of TGF-β signaling using neutralizing antibodies, we did not explore downstream signaling mechanisms in depth. Whether MDSCs interact with HSCs through other soluble factors or exosome-mediated communication requires further investigation.

**Figure 5:**
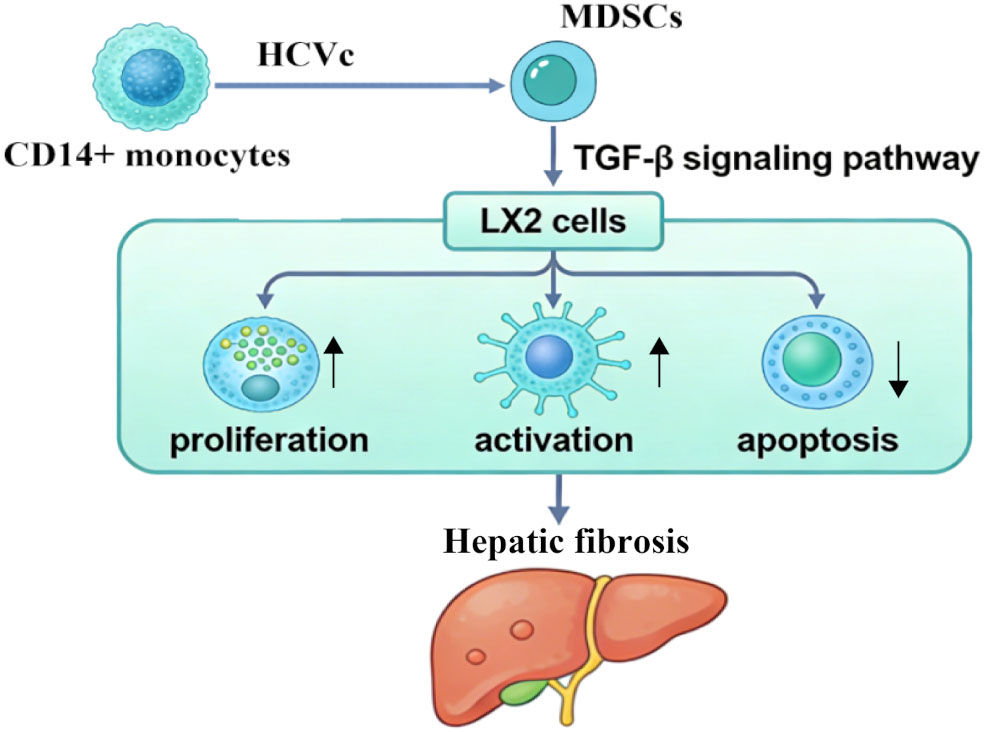
Schematic diagram of the mechanism by which hepatitis C virus core protein-induced myeloid-derived suppressor cells promote hepatic fibrosis by regulating hepatic stellate cell function via TGF-β.

In summary, this study reveals a novel immunopathological mechanism whereby HCVc exacerbates liver fibrosis. Specifically, HCVc induces myeloid-derived suppressor cells (MDSCs), which activate LX2 via the TGF-β signaling pathway, thereby driving liver fibrosis through this novel pathway (**Figure 5**). This discovery positions viral proteins, immunosuppressive cells, and fibrotic effector cells along a single pathological axis—”HCVc–MDSCs–TGF-β–LX2”—providing an integrated perspective for understanding the progression of HCV-associated liver disease.

## Data Availability

The raw data supporting the conclusions of this article will be made available by the authors, without undue reservation.
